# S47. Tumour infiltrating lymphocytes - a predictive marker?

**DOI:** 10.1186/2051-1426-2-S2-I10

**Published:** 2014-03-12

**Authors:** J Galon

**Affiliations:** 1 INSERM U872, Cordeliers Research Center, Paris, France

## Introduction

To date the anatomic extent of tumor (TNM classifications) has been by far the most important factors to predict the prognosis of cancer patients. However, this classification provides limited prognostic information in estimating the outcome in cancer and does not predict response to therapy.

## Materials and methods

large-scale approaches, and quantitative measurements, we evaluated the importance of the host-immune response within human tumors.

## Results

We showed that tumors from human colorectal cancer with a high density of infiltrating memory and effector memory T-cells (TEM) are less likely to disseminate to lymphovascular and perineural structures and to regional lymph-nodes. We showed that the combination of immune parameters associating the nature, the density, the functional orientation and the location of immune cells within the tumor was essential to accurately define the impact of the local host immune reaction on patients prognosis. We defined these parameters as the “immune contexture”, and factors modulating it will be discussed. Based on the *immune contexture*, a standardized, simple and powerful immune stratification system, termed the *“Immunoscore”*, was delineated that may bear a prognostic power superior to that of the currently used cancer staging system. Tumor invasion parameters were statistically dependent on the host-immune reaction. A worldwide Immunoscore consortium is testing the prognostic value of the Immunoscore, using a standardized assay to routinely measure the immune status of a cancer patient.

## Conclusions

The functional orientation of the *immune contexture* is characterized by immune signatures qualitatively similar to those predicting response to immunotherapy, Thus, the continuum of immune response existing, spanning a balance between tumor cell growth and elimination, will be discussed.

